# Loose-fit polypseudorotaxanes constructed from γ-CDs and PHEMA-PPG-PEG-PPG-PHEMA

**DOI:** 10.3762/bjoc.10.257

**Published:** 2014-10-23

**Authors:** Tao Kong, Lin Ye, Ai-ying Zhang, Zeng-guo Feng

**Affiliations:** 1 School of Materials Science and Engineering, Beijing Institute of Technology, Beijing 100081, China

**Keywords:** block copolymer, γ-CD, loose-fit, poly(2-hydroxyethyl methacrylate), polypseudorotaxane

## Abstract

A pentablock copolymer was prepared via the atom transfer radical polymerization of 2-hydroxyethyl methacrylate (HEMA) initiated by 2-bromoisobutyryl end-capped PPO-PEO-PPO as a macroinitiator in DMF. Attaching PHEMA blocks altered the self-assembly process of the pentablock copolymer with γ-CDs in aqueous solution. Before attaching the PHEMA, the macroinitiator was preferentially bent to pass through the inner cavity of γ-CDs to give rise to tight-fit double-chain stranded polypseudorotaxanes (PPRs). After attaching the PHEMA, the resulting pentablock copolymer was single-chain stranded into the interior of γ-CDs to form more stable, loose-fit PPRs. The results of ^1^H NMR, WXRD, DSC, TGA, ^13^C CP/MAS NMR and FTIR analyses indicated that γ-CDs can accommodate and slip over PHEMA blocks to randomly distribute along the entire pentablock copolymer chain. This results in unique, single-chain stranded PPRs showing no characteristic channel-type crystal structure.

## Introduction

Cyclodextrins (CDs) are a family of cyclic oligosaccharides composed of 6, 7 or 8 glucose units linked via α-1,4-glycosidic bonds. Due to the presence of a hydrophobic inner cavity with different geometric dimensions, CDs can act as host molecules to not only small guest molecules, but also to linear polymeric guest molecules. They can self-assemble into novel inclusion complexes (ICs), or polypseudorotaxanes (PPRs) and polyrotaxanes (PRs) end-capped by bulky stoppers. For example, α-CDs typically include PEG, but not PPG, β-CDs contain PPG instead of PEG, and γ-CDs accommodate either single-chain PPG or double-chain PEG [1]. The driving force behind the self-assembly is mostly ascribed to a suitable fit between the cross-sectional area of the incoming polymer chain and the cavity size of the CDs [2]. However, the cavity shape and size of CDs are deformable and variable to some extent. Their stability can vary depending on the solution, incoming guest molecules, and especially the number of glucose units (i.e., more glucose units give way to more structure flexibility) [3]. This possibly explains how Harada et al. [4] reported the first PPRs (comprised of γ-CD and PEG) as early as the 1990s. The γ-CD-based PPRs with designated supramolecular structure have been seldom prepared as compared with the α-CD- or β-CD-based PPRs [5–13].

**Scheme 1:**
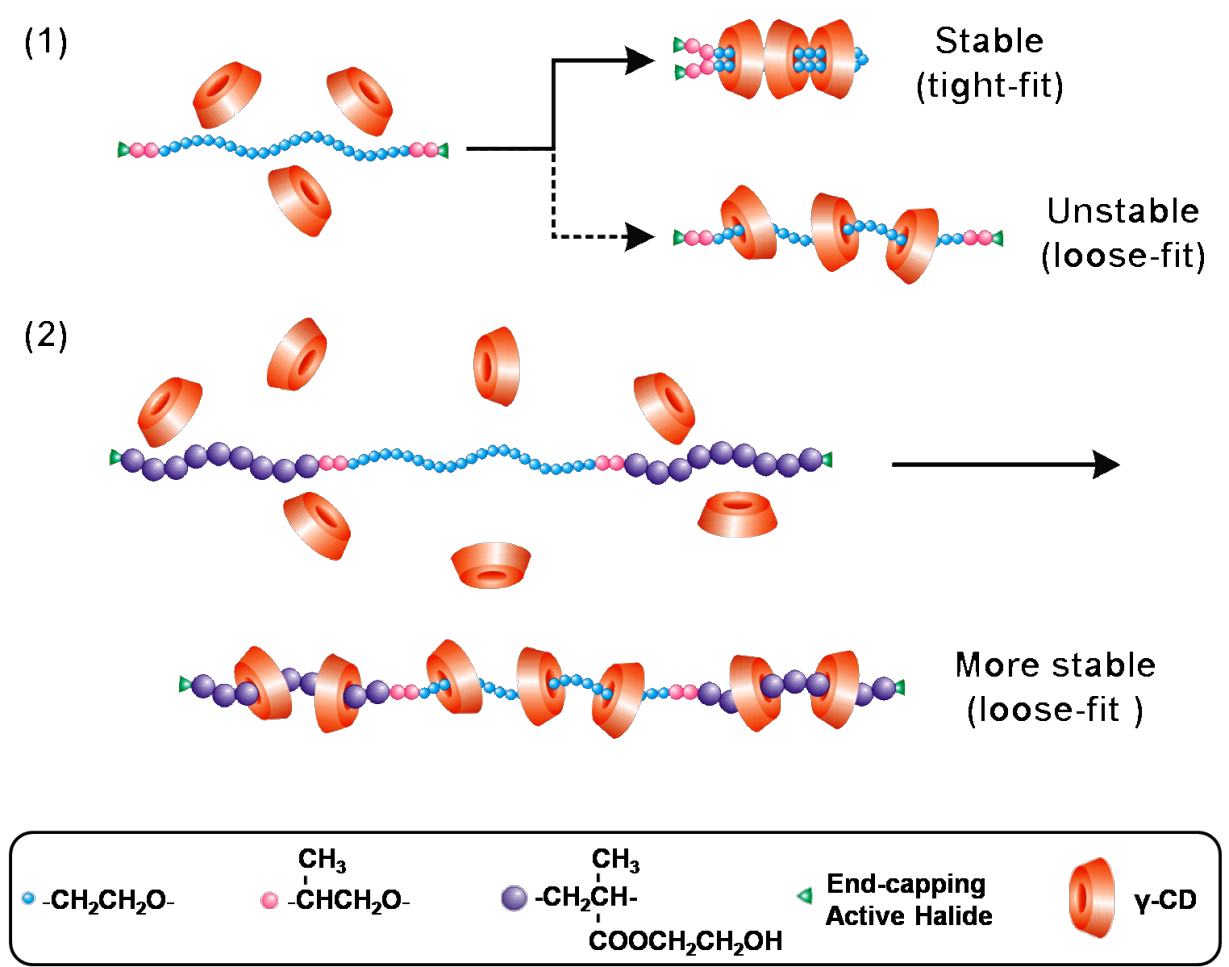
Schematic description of self-assembly of γ-CDs with BrPPO-PEO-PPOBr and PHEMA-PPO-PEO-PPO-PHEMA in aqueous solution.

Besides the typical double-chain stranded PPR showing a characteristic channel-type crystal structure, as reported by Harada et al. [4], the so-called single-chain stranded γ-CD-based PPRs or PRs have recently attracted tremendous attention. Their potential smart material and biomedical applications stem from their unique loose-fit rather than tight-fit structure of γ-CDs with a guest polymer [5]. Due to their superior deformability and adaptability, γ-CDs are able to slip over the bulkier PNIPAAm homo- and co-polymers to give rise to single-chain stranded, loose-fit PPRs or PRs showing no characteristic channel-type crystal structure [14–16]. To the best of our knowledge, self-assembled PPRs from γ-CDs with the bulkier poly(2-hydroxyethyl methacrylate) (PHEMA)-flanked block copolymers have not yet been reported. Herein, a pentablock copolymer PHEMA-PPO-PEO-PPO-PHEMA is prepared via atom transfer radical polymerization (ATRP) in DMF, and allowed to self-assemble with γ-CDs in aqueous solution to form PPRs. The results of ^1^H NMR, WXRD, DSC, TGA, ^13^C CP/MAS NMR and FTIR analyses indicate that the attachment of PHEMA clearly changes the self-assembly direction of γ-CDs with PHEMA-PPO-PEO-PPO-PHEMA. This results in unique, single-chain stranded, loose-fit PPRs, instead of the PEG-bent, double-chain stranded, tight-fit ones as shown in Scheme 1.

## Results and Discussion

### Self-assembly of γ-CDs with a macroinitiator and a pentablock copolymer in aqueous solution

**Scheme 2:**
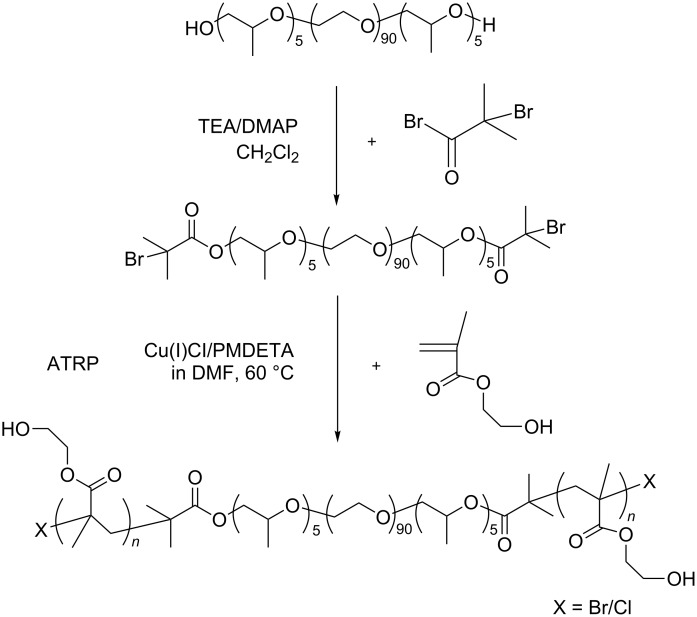
Synthetic pathway of a pentablock copolymer.

As previously reported, a distal 2-bromoisobutyryl end-capped PEG was preferably bent to fit into the cavity of γ-CDs to form stable and unconventionally conformational PPRs. γ-CDs can accommodate the bulkier poly(*N*-isopropylacrylamide) (PNIPAAm) blocks to give single-chain stranded, loose-fit PPRs or PRs [14–16]. To extend the scope of self-assembly of γ-CDs with polymers having bulkier cross-sectional areas, PHEMA is attached to both ends of PPO-PEO-PPO by ATRP to yield a pentablock PHEMA-PPO-PEO-PPO-PHEMA copolymer. It is then used to investigate the possibility of self-assembly with γ-CDs [17–18]. The synthetic pathway for the pentablock copolymer is shown in Scheme 2. To shed light on the impact of end-capping groups on the self-assembly direction of γ-CDs with an incoming polymer chain, a PPO-PEO-PPO, triblock copolymer was studied. The copolymer had an average degree of polymerization (DP) of five PPO flanking blocks instead of pure PEG in order to enlarge the volume of the end-capping, 2-bromoisobutyryl group. Furthermore, there is a hydrolytic side reaction of end-capped bromine in the in situ aqueous ATRP of NIPAAm that can reduce the chain end functionality and the efficiency of future chain end modification. Thus, Cu(I)Cl/PMDETA was chosen as catalyst and DMF as solvent for the ATRP of HEMA in this study [19].

Table 1 summarizes the ^1^H NMR and GPC analytical results of the pentablock copolymer. The high conversation ratio (87%) of the monomer and the low *M*_w_/*M*_n_ value (1.23) in the preparation of PHEMA-PPO-PEO-PPO-PHEMA suggest that the ATRP carried out using Cu(I)Cl/PMDETA as a catalyst and DMF as a solvent was successful (Supporting Information, Figure S1 and Figure S2). For convenience, the resulting macroinitiator was designated as BrPEPBr and the pentablock copolymer as PEP26M.

**Table 1:** Composition, molecular weight and molecular weight distribution of the pentablock copolymer.

	Reaction time/h	Molar ratio of BrPEPBr:HEMA		*M*_n_^a^	*M*_n_^b^	*M*_w_/*M*_n_^b^
		Feed ratio	Found ratio^a^			

PEP26M	24	1:30	1:26.0	8260	9.0 × 10^3^	1.23

^a^Determined by ^1^H NMR analysis in DMSO-*d*_6_. ^b^Determined by GPC analysis.

**Figure 1:**
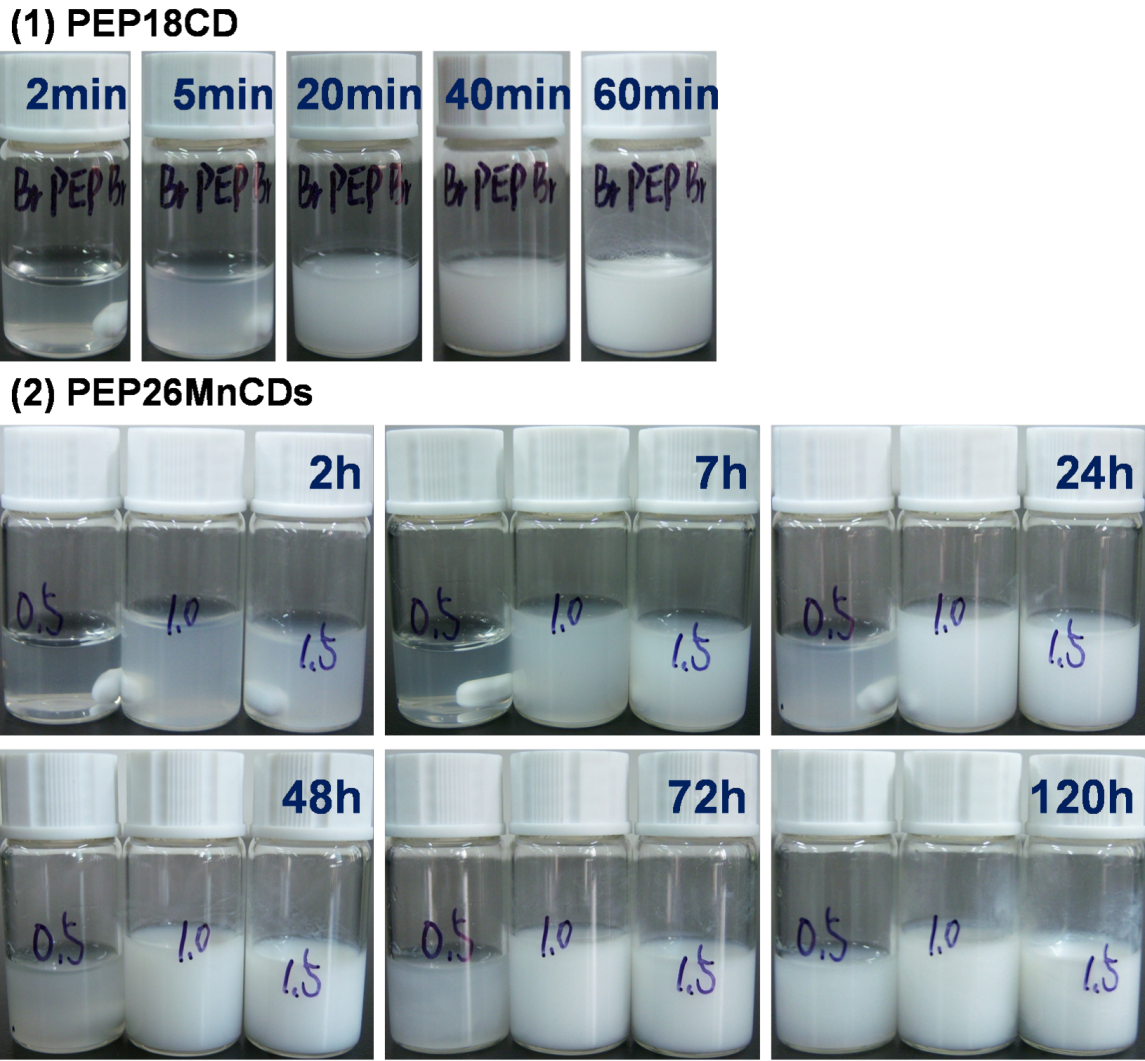
Photographs of the turbidity evolution in PEP18CD (1) and PEP26MnCDs (2) (PEP26M9CD (left), PEP26H18CD (middle) and PEP26M27CD (right)) at room temperature.

As illustrated in Scheme 1, both double-chain stranded, tight-fit and single-chain stranded, loose-fit PPRs are constructed from the self-assembly of γ-CDs with the macroinitiator and the pentablock copolymer in aqueous solution at room temperature. The PPRs obtained from the inclusion complexation of γ-CDs with PHEMA-PPO-PEO-PPO-PHEMA are assigned as PEP26MnCD, where n represents the feed molar ratio of γ-CD to PEP26M. Meanwhile, BrPEPBr was also self-assembled with γ-CDs (feed molar ratio of BrPEPBr/γ-CD = 1:18) in aqueous solution to provide a reference PPR labelled as PEP18CD. The theoretical and resulting compositions and yields of PEP26MnCDs and PEP18CD are summarized in Table 2. The evolution of the self-assembly of γ-CDs with the macroinitiator and pentablock copolymer is depicted in Figure 1. As can be seen, the turbidity of the PEP18CD solution is abruptly increased within several minutes. This is in contrast to several hours for PEP26MnCDs, which is consistent with the conformational differences in the double-chain and single-chain stranded PPRs. This implies that PEG tends to be bent in order to quickly pass through the inner cavity of γ-CDs when the 2-bromoisobutyryl initiating groups are attached to two ends. However, if these end-capping groups were replaced by the bulkier polymer blocks (e.g., PHEMA), the self-assembly of γ-CDs would become a time-consuming process. This is because the γ-CDs would need to accommodate and slip off the bulkier PHEMA blocks in order to distribute along the whole polymer chain, leading to the more stable, single-chain stranded, loose-fit PPRs.

**Table 2:** Theoretical and resulting compositions and yields of PPRs.

	Linear guest molecule	Molar ratio of guest molecule:γ-CD		Yield^b^
		Feed ratio	Resulting ratio^a^	

PEP26M9CD	PEP26M	1:9	1:10.7	22.5%
PEP26M18CD		1:18	1:16.0	43.0%
PEP26M27CD		1:27	1:21.9	45.1%
PEP18CD	BrPEPBr	1:18	21.3	72.9%

^a^Determined by ^1^H NMR analysis in DMSO-*d*_6_. ^b^Yield is calculated based on the weight of precipitated PPR divided by that of all the feed materials.

Furthermore, as seen in Figure 1, the turbidity of the PEP26MnCD solutions increases rapidly with the increase in the feed molar ratio of γ-CDs to PEP26M. This suggests that more γ-CDs entrap the PEP26M main chain and a faster accommodation process is accomplished in aqueous solution. The resulting molar ratio of γ-CDs to PEP26M increased from 10.7 to 21.9 when the feed molar ratio was increased from 9 to 27, respectively. Although all of the yields in the range of 22.5% to 45.1% appear low in this study, they are markedly higher than ever reported for single-chain stranded, loose-fit, CD-based PPRs or PRs [5,14–16]. The significant difference in yield between PEP26M18CD and PEP18CD reveals another demonstration of the reliable diversity in the loose-fit and tight-fit PPRs.

### Characterization of PPRs self-assembled from γ-CDs with a macroinitiator and pentablock copolymer

**Figure 2:**
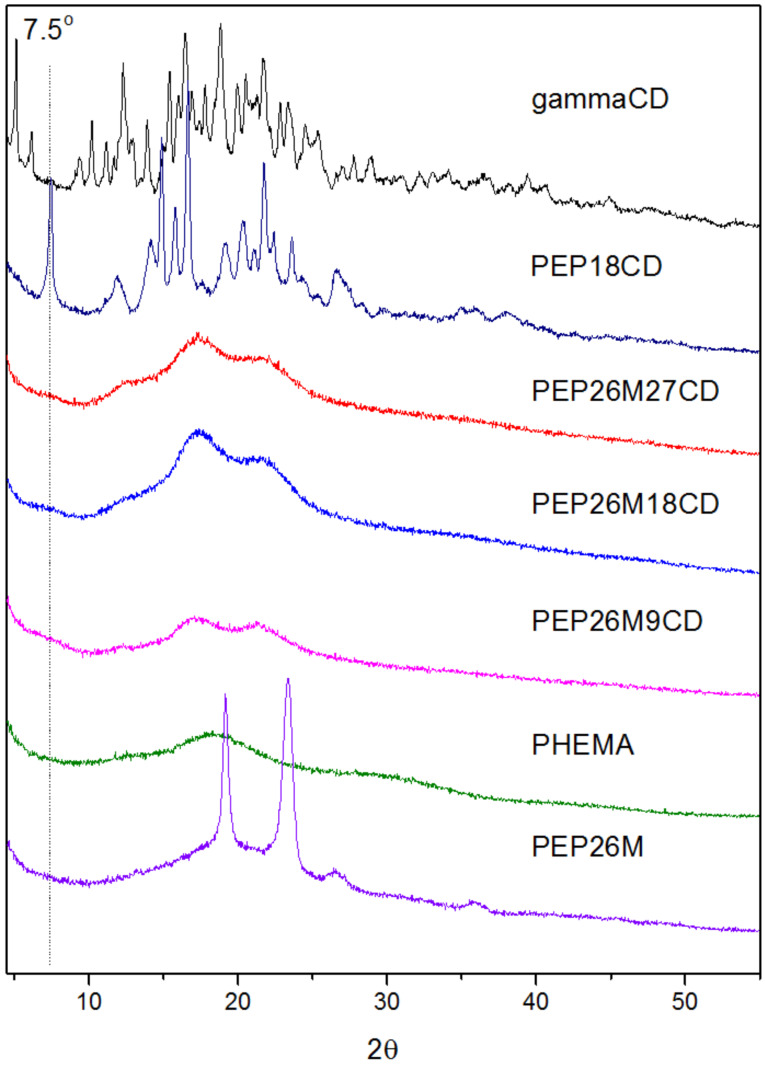
WXRD spectra of γ-CD, PHEMA, PEP26M, PEP18CD and PEP26MnCDs.

The WXRD patterns of γ-CD, PEP26M, PHEMA and PPRs are presented in Figure 2. The major diffraction peaks of γ-CD appear at 2θ = 5.1°, 10.2°, 12.3°, 15.4°, 16.4°, 18.8° and 21.7°, respectively, which correspond to a cage-type crystal structure [20]. The pentablock polymer PEP26M shows two strong peaks at 19.2° and 23.3°, originating from the crystal structure of the PEO central block [21]. The pure PHEMA displays two broad-featured peaks at 2θ = 18.3°, 29.2° that are characteristic of an amorphous polymer. As portrayed in Scheme 1, BrPEPBr is apt to form a stable, single PEO-bent conformation in order to self-assemble with γ-CDs in aqueous solution. This shows a characteristic, channel-type, crystal structure diffraction peak at 2θ = 7.5° in accordance with our previous reports [22]. Interestingly, all of the samples resulting from the self-assembly of γ-CDs with PEP26M reveal three new broad peaks at 2θ = 12.4°, 17.3° and 21.5°, but no diffraction peak at 7.5°. This unique diffraction pattern is quite similar to that of the single-chain stranded, loose-fit γ-CD-based PPRs or PRs as previously reported [5,14–16,23].

Due to a relatively bulkier yet appropriate cross-sectional area, the attachment of PHEMA evidently changes the self-assembly direction of γ-CDs with the pentablock copolymer as compared with the macroinitiator. In the case of self-assembly with the latter, the two parallel macroinitiator chains are not permitted to pass through the cavity of γ-CDs, and alternatively, a single PEG chain is bent into a double-chain strand. This gives rise to a tight-fit PPR exhibiting a characteristic channel-type crystal structure. However, for the self-assembly with the pentablock copolymer, an appropriately-sized chain of HEMA repeating units enables PHEMA to be included into and to penetrate through γ-CDs. This yields more stable, single-chain stranded, loose-fit PPRs showing no characteristic channel-type crystal structure.

**Figure 3:**
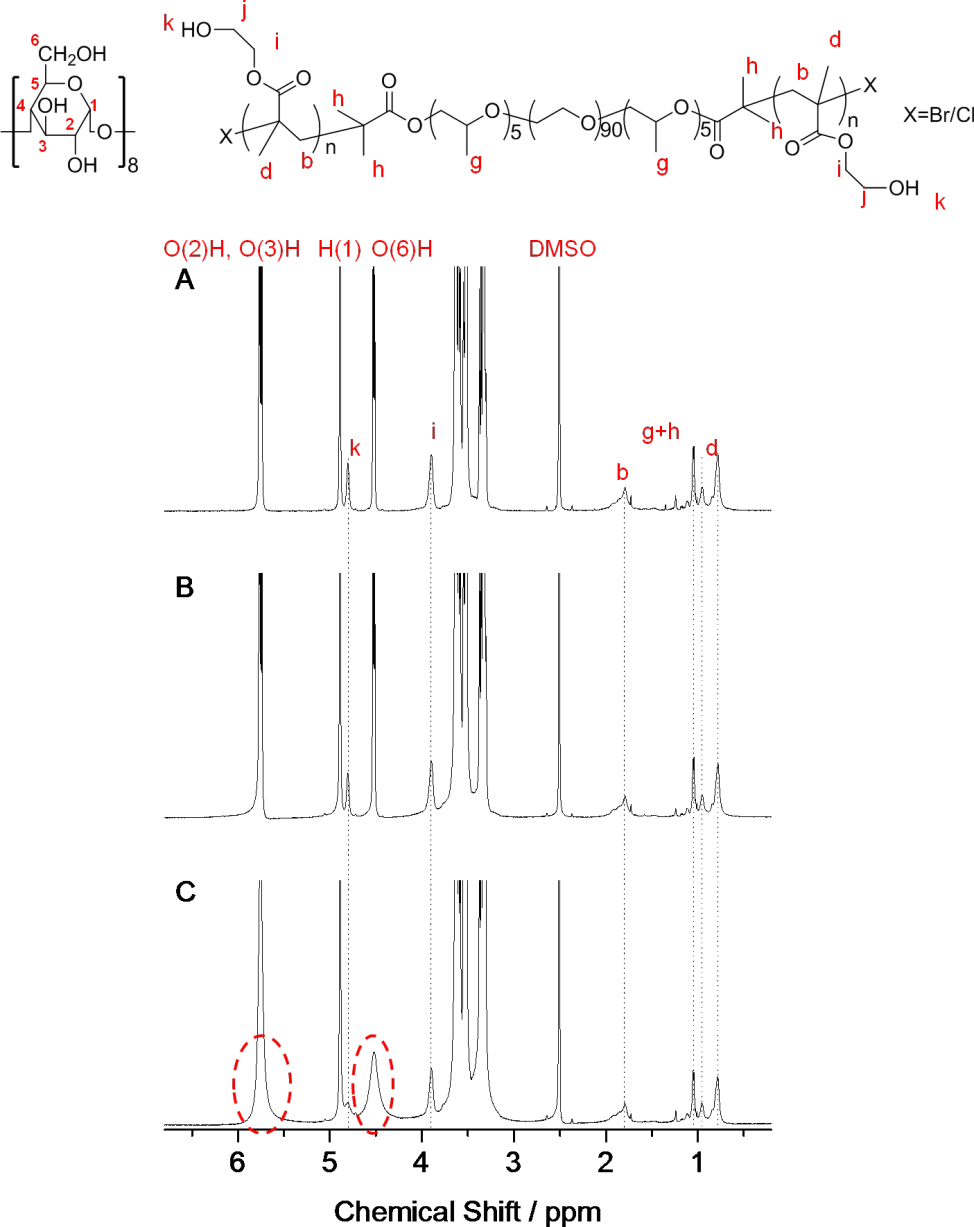
^1^H NMR spectra of PEP26M9CD (A), PEP26M18CD (B) and PEP26M27CD (C).

This single-chain stranded, loose-fit structure is also supported by ^1^H NMR analysis. According to previous reports [12,24], the inside cavity of each γ-CD molecule could accommodate two PO or 2.2 vinyl repeating units into the resulting single-chain stranded PPRs. As outlined in Table 2, both the PHEMA and PPO blocks in PEP26M would theoretically be covered by about 16.8 (10/2 + 26/2.2) γ-CD molecules, which is less than the resulting molar ratio of 21.9 for PEP26M27CD. This is most likely caused by γ-CDs slipping into the middle PEO block. Additionally, as seen in Figure 3C, the hydroxy group resonance peaks (O(2)H, O(3)H and O(6)H) of γ-CD in PEP26M27CD are clearly broader as compared with PEP26M9CD and PEP26M18CD. This is due to the decrease in conformational flexibility upon PPR formation. Furthermore, this indicates that a number of γ-CDs can still be held on the pentablock copolymer axle at a higher feed molar ratio, even in a highly polar DMSO solvent as previously described [25].

**Figure 4:**
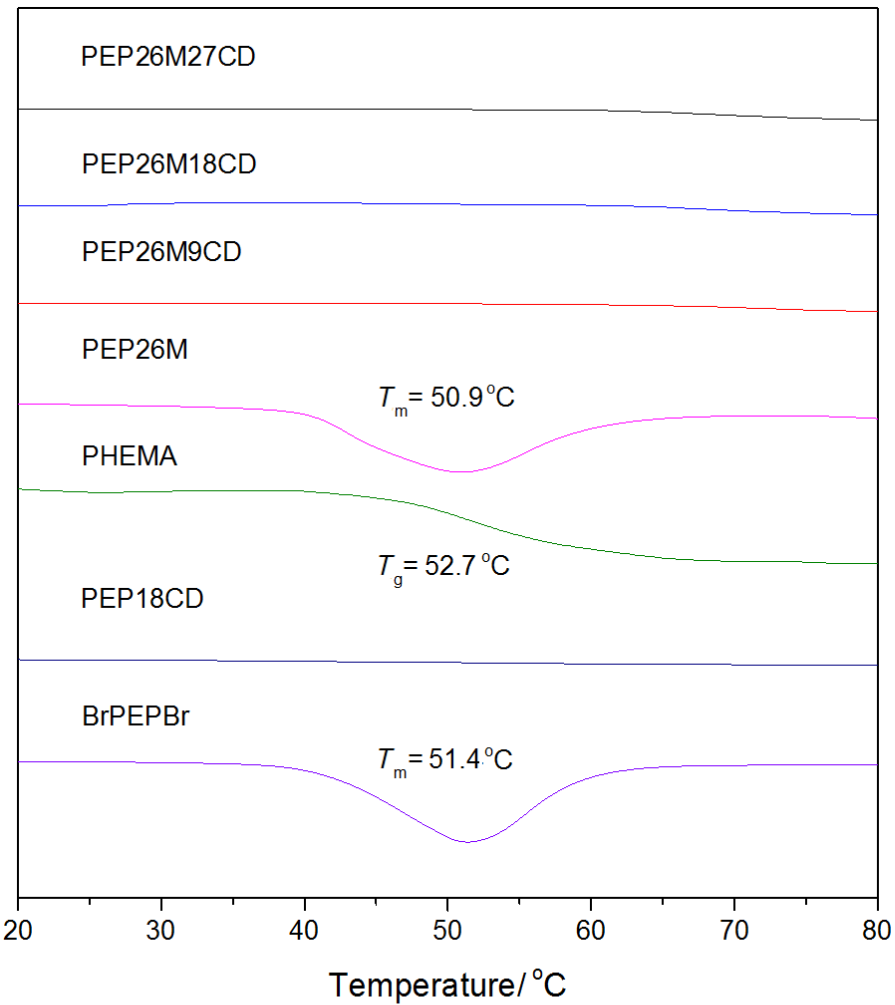
DSC curves of PHEMA, PEP26M, PEP26MnCDs, BrPEPBr and PEP18CD.

DSC measurements provide further evidence of the unique, single-chain stranded, loose-fit structure formed by the self-assembly of γ-CDs with PHEMA-PPO-PEO-PPO-PHEMA, as seen in Figure 4. These results clearly show endothermic peaks in PEP26M and BrPEPBr (in addition to a glass transition region in PHEMA), which correspond to the melting temperature (*T*_m_) of the crystallized PEO segment and the glass transition temperature (*T*_g_) of pure PHEMA, respectively. On the contrary, the curves of the PEP26MnCDs samples exhibit no obvious signal mutation from 20 to 80 °C. This indicates that either both PEO and PHEMA blocks stay amorphous or that the thermal motion of chain segments in PEO and PHEMA blocks are roughly restricted due to inclusion into the cavity of γ-CDs. Owing to the formation of a single PEO-bent conformation with γ-CDs, the corresponding endothermic peak of PEO is also absent, as evidenced in PEP18CD.

**Figure 5:**
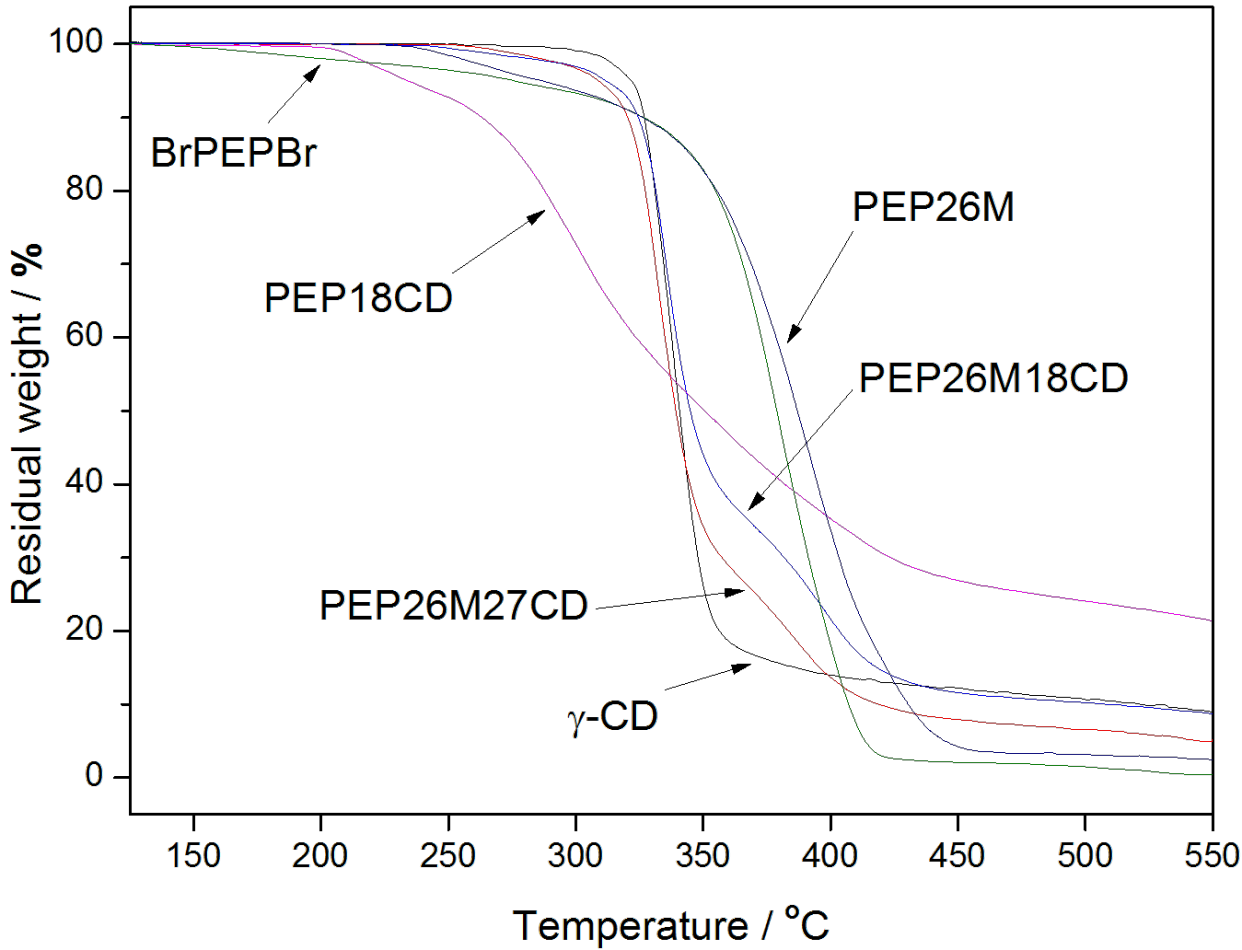
TGA curves of γ-CD, PEP26M, PEP26MnCDs, BrPEPBr and PEP18CD.

TGA analysis also demonstrates the single-chain stranded, loose-fit structure of PEP26MnCDs, as depicted in Figure 5. Attributed to the protection of the γ-CD cover, the initial temperature of thermal weight loss for PEP26M27CD occurs at 260 °C, which is much higher than 225 °C for uncovered PEP26M. After the thorough thermal decomposition of covering γ-CD molecules, namely above 360 °C, PEP26M27CD undergoes another distinct thermal weight loss. This might be assigned to the decomposition of residual pentablock copolymer. Conversely, as compared with the pure γ-CD, PEP26M27CD starts to decompose at a relatively lower temperature and at a slower rate. This is similar to the behavior of the loose-fit PPR structure in which the entrapped γ-CDs are stacked in a less-ordered non-crystalline structure. The TGA curve of PEP26M18CD exhibits a similar trend. Additionally, in addition to the same shift in the initial temperature of thermal weight loss (arising from stranded γ-CDs), PEP18CD displays a unique, higher, residual weight ratio (>20%) at 550 °C, which might be credited to its tight-fit supramolecular structure.

**Figure 6:**
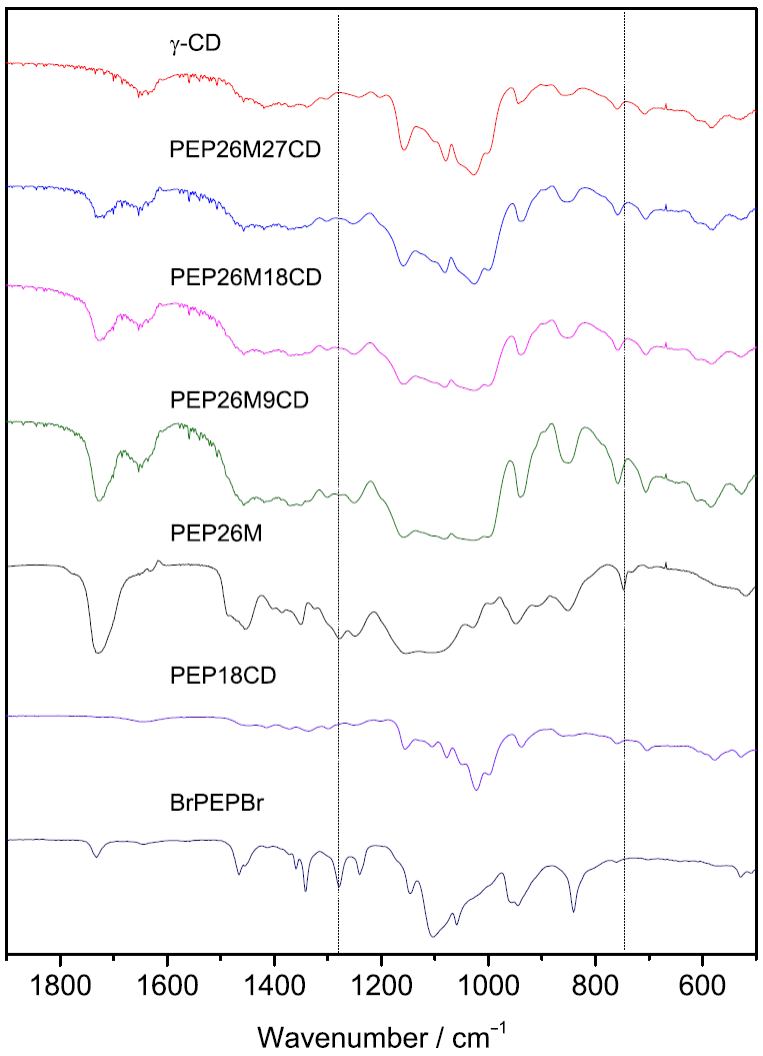
FTIR spectra of γ-CD, PEP26M, PEP18CD and PEP26MnCDs.

FTIR spectroscopy analysis is a powerful technique to highlight the supramolecular structure of host and guest molecules in PPRs [26]. The FTIR spectra of PEP26MnCDs and their precursors are given in Figure 6. The spectrum of PEP26M exhibits distinct vibrational peaks around 750 cm^−1^ (out of plane bending of C–O in the ester of HEMA repeat units) [27] and 1280 cm^−1^ (CH_2_ twist in EO repeat units) [28]. Importantly, both peaks vanish in the spectra of PEP26MnCDs, which is characteristic of the restricting and shielding effects from the inner cavity of γ-CDs to the vibrations of correlative chemical bonds. This offers supplementary (if not direct) proof of the alleged single-chain stranded, loose-fit structure of PEP26MnCDs. Similarly, PEP18CD also confirms the expected disappearance of the CH_2_ twist vibrational peak, which occurs when γ-CDs compactly locate on the PEO chain in a head–head or tail–tail manner.

**Figure 7:**
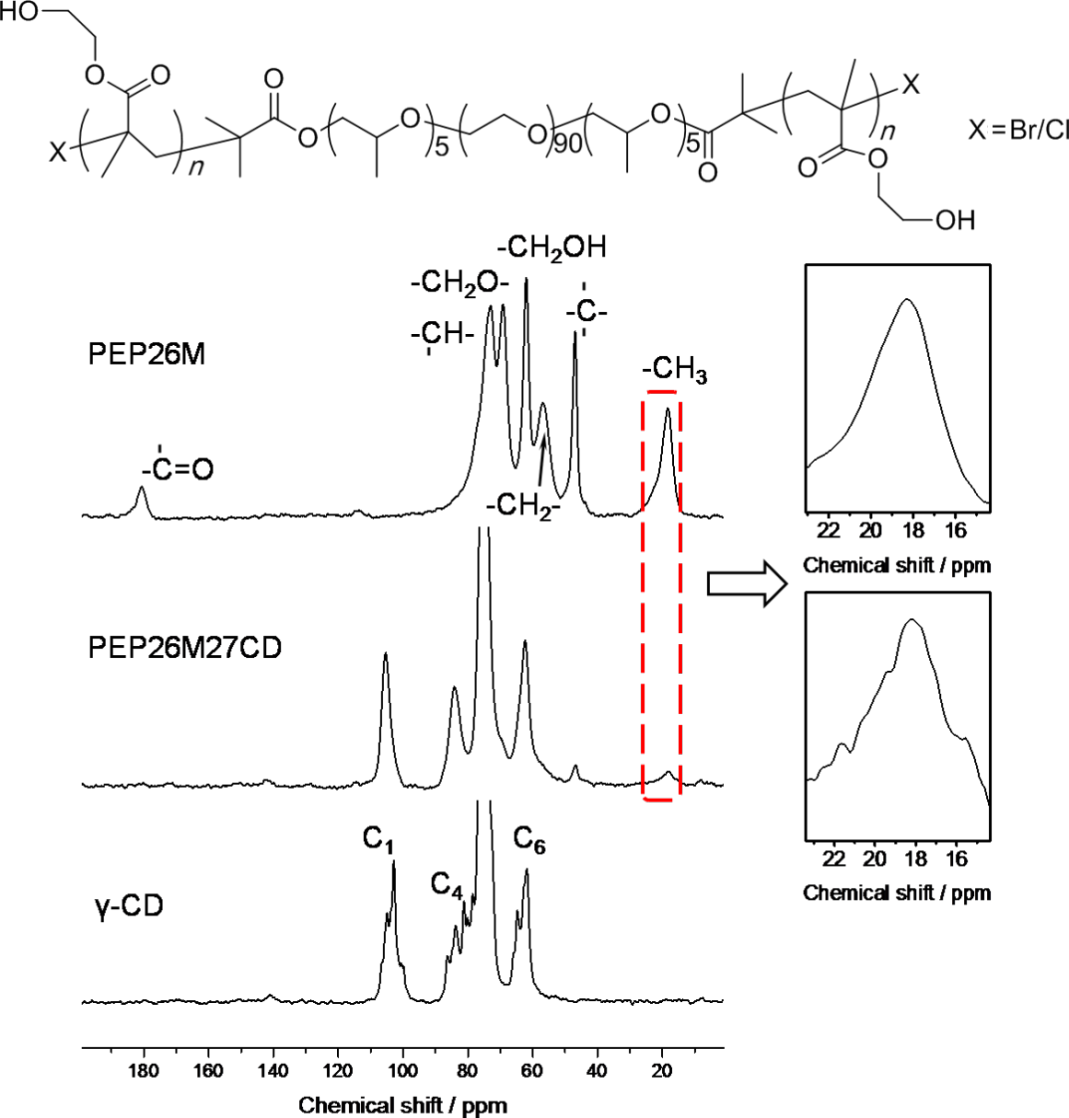
^13^C CP/MAS NMR spectra of PEP26M, γ-CD and PEP26M27CD.

Figure 7 compares the ^13^C CP/MAS NMR spectrum of PEP26M27CD with those of PEP26M and γ-CD. Consistent with previous research [29–30], uncomplexed γ-CDs assert less symmetrical cyclic conformations with clear multiple C_1_, C_4_ and C_6_ resonance peaks. This is in contrast to single C_1_, C_4_ and C_6_ resonances in PEP26M27CD, which prove that more symmetrical, cyclic γ-CDs cover and reside along the PEP26M main chain of PEP26M27CD. Noteworthy is the fact that the –CH_3_ resonance of PEP26M27CD similarly displays weak peak splits. This implies that the PHEMA and PPO blocks probably adopt unusual substructure conformations in the cavity of γ-CDs, different from the general morphology in the original PEP26M. As a result, they create the single-chain stranded, loose-fit structured PPRs. A further investigation focusing on molecular recognition between γ-CD and PHEMA is ongoing in our laboratory.

## Conclusion

A series of γ-CD-based PPRs were prepared via the self-assembly of γ-CDs with a pentablock copolymer PHEMA-PPO-PEO-PPO-PHEMA in aqueous solution at room temperature. The resulting PPRs possess a unique single-chain stranded, loose-fit conformation showing no characteristic channel-type crystal structure. This finding highlights a novel model to fulfil molecular recognition between transformable γ-CDs and functional, bulkier vinyl polymers. This highlights the potential for smart material and biomedical applications. Further investigations are underway in our laboratory.

## Experimental

### General measures

^1^H NMR (400 MHz) spectra were recorded on a Bruker ARX-400 spectrometer at room temperature using DMSO-*d*_6_ as a solvent and tetramethylsilane (TMS) as an internal standard. ^13^C cross-polarization magic-angle spinning (CP/MAS) NMR spectra were measured on a Bruker AV 300 NMR spectrometer with a single contact time of 1 ms and a spinning rate of 5 kHz. Chemical shifts were compared to an external adamantane standard. Gel permeation chromatographic (GPC) measurements were carried out at 40 °C on a HLC-8320GPC (TOSOH, Japan) instrument using THF as eluent at a flow rate of 0.3 mL/min. All of the GPC data were calibrated using polystyrene (PS) standards. Fourier transform infrared spectroscopy (FTIR) spectra were measured using a Shimadzu IR Prestige-21 FTIR spectrometer at room temperature in the range between 4000 and 500 cm^−1^, with a resolution of 2 cm^−1^ and 25 averaged scans. Samples were prepared by mixing with dry KBr powder. Differential scanning calorimetry (DSC) measurements were run on a SHIMADZU DSC-60 differential scanning calorimeter with a scan temperature range from 20 to 80 °C at a scan rate of 10 °C min^−1^ and purged with nitrogen. The samples were encapsulated in hermetically-sealed aluminum pans, and underwent two, 20 to 80 °C heating procedures. Data were collected during the second heating run. TGA was performed with a TA SDT 2960 instrument at a heating rate of 10 °C min^−1^ while purged with nitrogen, and the temperature was scanned from ambient temperature to 550 °C. Wide-angle X-ray diffraction (WXRD) measurements were carried out with powder samples using a Shimadzu XD-D1 X-ray diffractometer. The radiation source was Ni-filtered, Cu Kα radiation with a wavelength of 0.154 nm. The voltage was set to 40 kV and the current to 40 mA. Samples were placed on a sample holder and scanned from 2θ = 4.5 to 60 ° at a speed of 5 ° min^−1^.

### Materials

γ-CD (Wako, Japan) and PPO-PEO-PPO (comprised of a central block of 90 PEO units and two flank blocks of 5 PO units having *M*_n_ = 4580 (Zhejiang Huangma Chemical Industry Group Co., Ltd, China)) were used as received without further purification. 2-Hydroxyethyl methacrylate (HEMA) (TCI, Japan) was passed over a short basic alumina column to remove the inhibitor before polymerization. *N*,*N*,*N*’,*N*”,*N*”-penta-methyldiethylenetriamine (PMDETA) and ethyl 2-bromoisobutyrate were purchased from Sigma, USA. Both 2-bromoisobutyryl bromide and 4-dimethylaminopyridine (DMAP) were available from Alfa Aesar, USA. Triethylamine (TEA) (VAS Chemical Reagents Company, China) was refluxed with *p*-toluenesulfonyl chloride and distilled under vacuum. Copper(I) chloride (Cu(I)Cl) was prepared from CuCl_2_, purified by stirring in hydrochloric acid, washed with methanol and finally dried under vacuum prior to use. CH_2_Cl_2_ was stirred with CaH_2_ and distilled under reduced pressure. DMF was supplied by Sinopharm Chemical Reagent Company, China and used without further purification. All other solvents and reagents were of analytical grade.

### Synthesis of 2-bromoisobutyryl end-capped PPO-PEO-PPO (BrPEPBr)

PPO-PEO-PPO was converted to the corresponding ATRP macroinitiator through the end-capping reaction with a fourfold molar excess of 2-bromoisobutyryl bromide in CH_2_Cl_2_. PPO-PEO-PPO (9.16 g, 2 mmol), DMAP (488 mg, 4 mmol) and TEA (404 mg, 4 mmol) were dissolved in 20 mL CH_2_Cl_2_ in a 100 mL three-neck round-bottom flask. Thereafter, another 20 mL of CH_2_Cl_2_ containing 2-bromoisobutyryl bromide (1.00 mL, 8 mmol) was added drop-wise under nitrogen. The reaction continued for 2 h at 0 °C and then for another 24 h at room temperature under stirring. Finally, the mixture was filtered to remove the precipitated bromide salt. The product was purified by precipitation into 500 mL of anhydrous ether at 10 °C. The sequence was repeated three times. ^1^H NMR analysis indicated that the degree of esterification was >99%, and the yield was 83.4% (Figure S3, Supporting Information File 1).

### Synthesis of PHEMA-PPO-PEO-PPO-PHEMA via ATRP

A typical procedure for the synthesis of the PHEMA-PPO-PEO-PPO-PHEMA pentablock copolymer via ATRP of HEMA was as follows. In a sealable Pyrex reactor, BrPEPBr (0.488 g, 0.1 mmol) was dissolved in 4 mL DMF. HEMA (0.39 g, 3.0 mmol) dissolved in 2 mL of DMF was added to this mixture, and then PMDETA (69.3 mg, 0.4 mmol) was added. The mixture was degassed by three freeze–pump–thaw cycles, then quenched in liquid nitrogen to which Cu(I)Cl (39.6 mg, 0.4 mmol) was added. The reactants in the reactor were degassed three times by purging with nitrogen. The reactor was sealed under vacuum and the reaction was maintained for 24 h at 60 °C under stirring. The polymerization was stopped after breaking the Pyrex reactor, and the product was dialyzed using a cellulose membrane (molecular weight cut-off (MWCO) = 3500) and lyophilized, resulting in a yield of 84.3%.

### Synthesis of PHEMA via ATRP

As previously reported [31], PHEMA (DP = 29, PDI = 1 .19) was synthesized by ATRP of HEMA in a DMF/H_2_O (w/w = 1:1) mixture at 25 °C using ethyl 2-bromoisobutyrate as an initiator, Cu(I)Cl as a catalyst and PMDETA as a ligand.

### Preparation of PPRs from the self-assembly of γ-CDs with a pentablock copolymer

A protocol for the preparation of PPRs built from the self-assembly of PHEMA-PPO-PEO-PPO-PHEMA with a varying amount of γ-CDs was as follows. A saturated aqueous solution containing a predetermined amount of γ-CDs was added to a 1.5 mL aqueous solution of the pentablock copolymer (0.10 g, 1.21 × 10^−2^ mmol), followed by vigorous stirring at room temperature for sufficient time to form a PPR. The resulting PPR was collected by centrifugation and washed with a small amount of water to remove residual free γ-CDs before freeze-drying.

## Supporting Information

Supporting Information File 1 contains a GPC trace of PEP26M and ^1^H NMR spectra of PEP26M and BrPEPBr.

File 1 Additional GPC trace and ^1^H NMR spectral data.
