# Supplementary material for: Loose-fit polypseudorotaxanes constructed from γ-CDs and PHEMA-PPG-PEG-PPG-PHEMA
Source: Beilstein J Org Chem. 2014 Oct 23;10:2461–9. doi: 10.3762/bjoc.10.257 (PMC4222382; doi:10.3762/bjoc.10.257)
Supplement: File 1 — Additional GPC trace and 1H NMR spectral data. [file Beilstein_J_Org_Chem-10-2461-s001.pdf]

Supporting Information

for

Loose-fit polypseudorotaxanes constructed from γ-CDs and PHEMA-PPG-PEG-PPG-PHEMA

Tao Kong, Lin Ye, Ai-ying Zhang and Zeng-guo Feng*

Address: School of Materials Science and Engineering, Beijing Institute of Technology, Beijing 100081, China

Email: Zeng-guo Feng - sainfeng@bit.edu.cn

*Corresponding author

Additional GPC trace and ^{1}H NMR spectral data

Content:

- GPC trace of PEP26M.
- ^{1}H NMR spectrum of PEP26M.
- ^{1}H NMR spectrum of BrPEPBr.

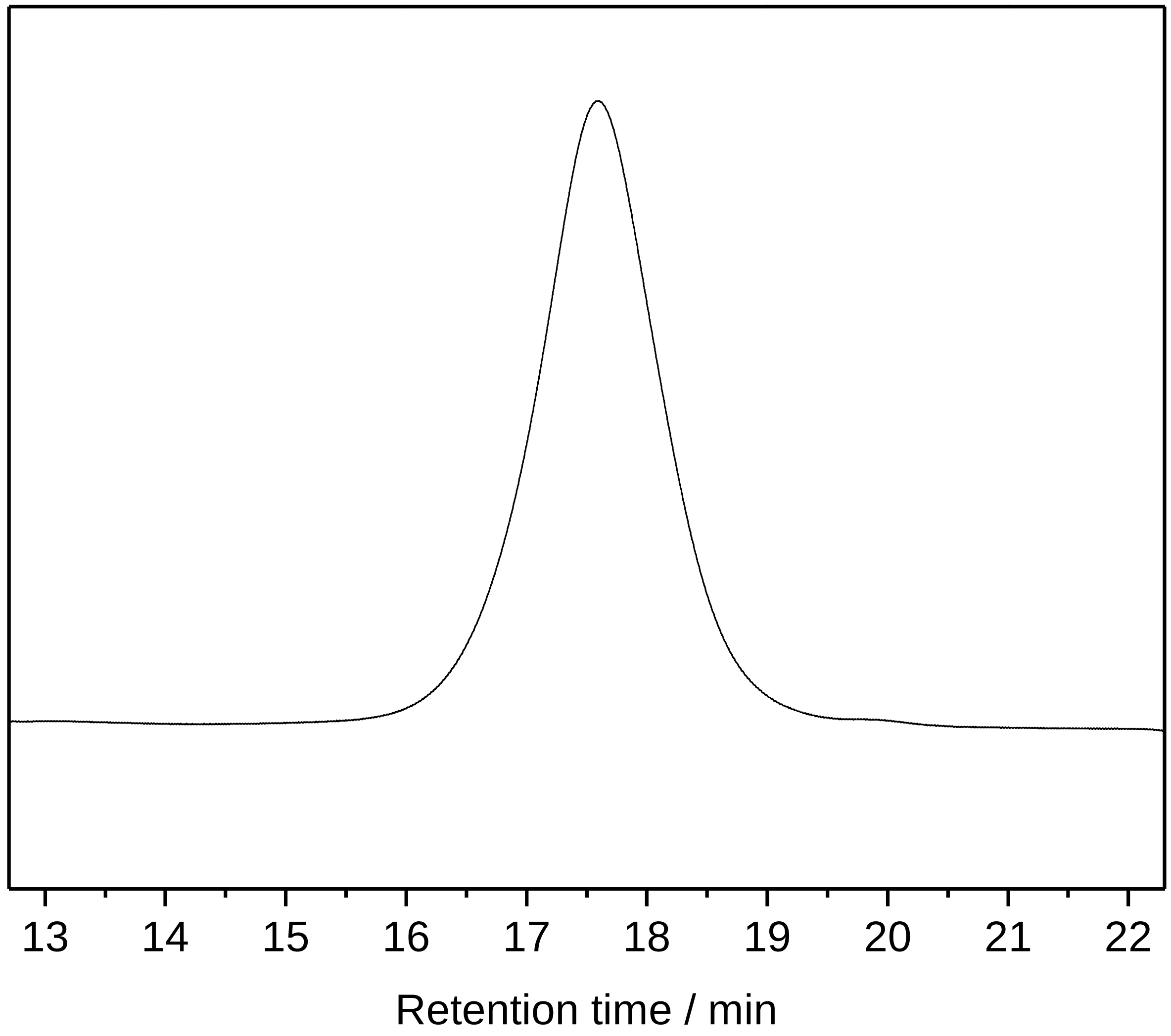

Figure S 1: GPC trace of PEP26M.

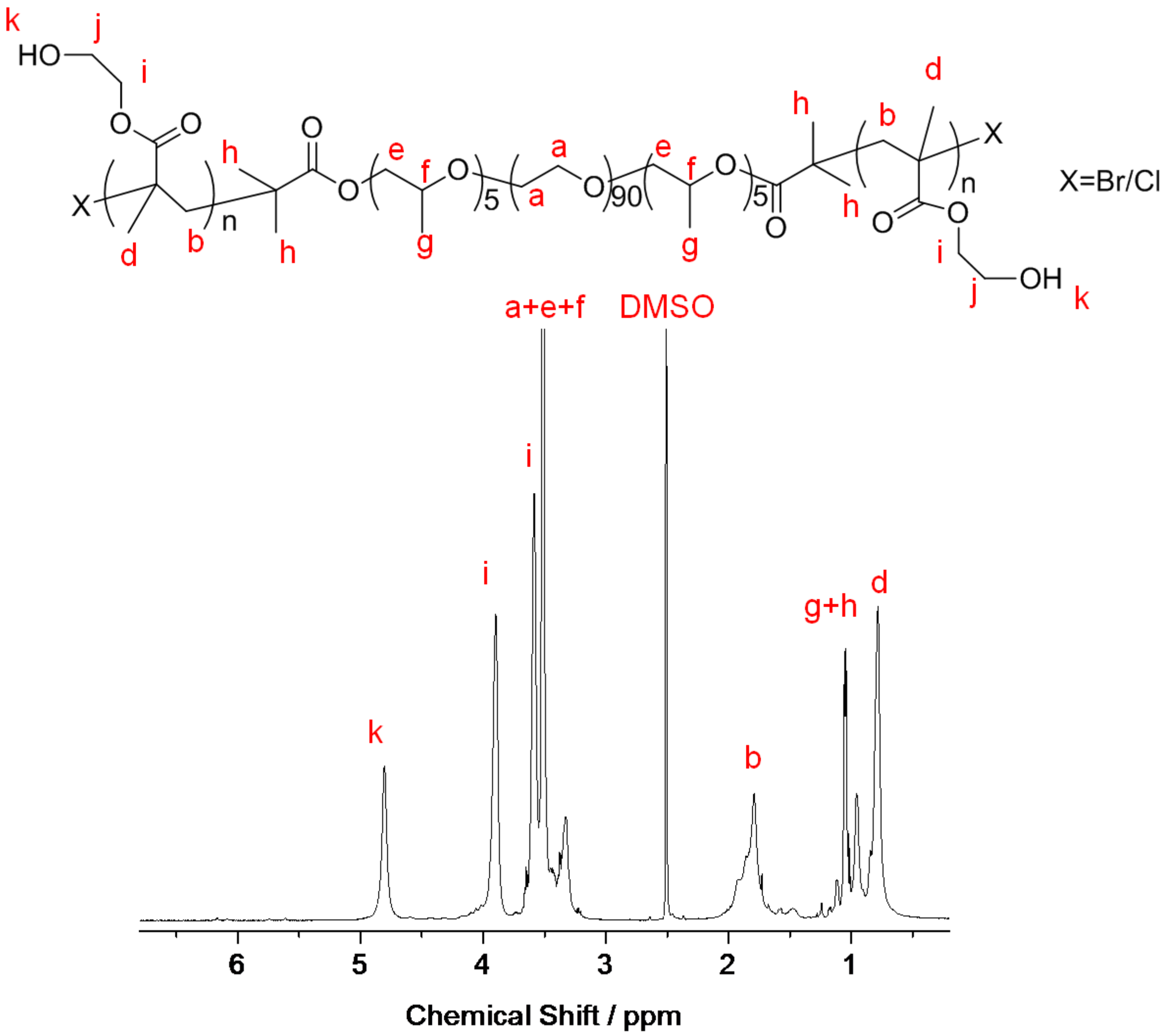

Figure S 2: ^{1}H NMR spectrum of PEP26M.

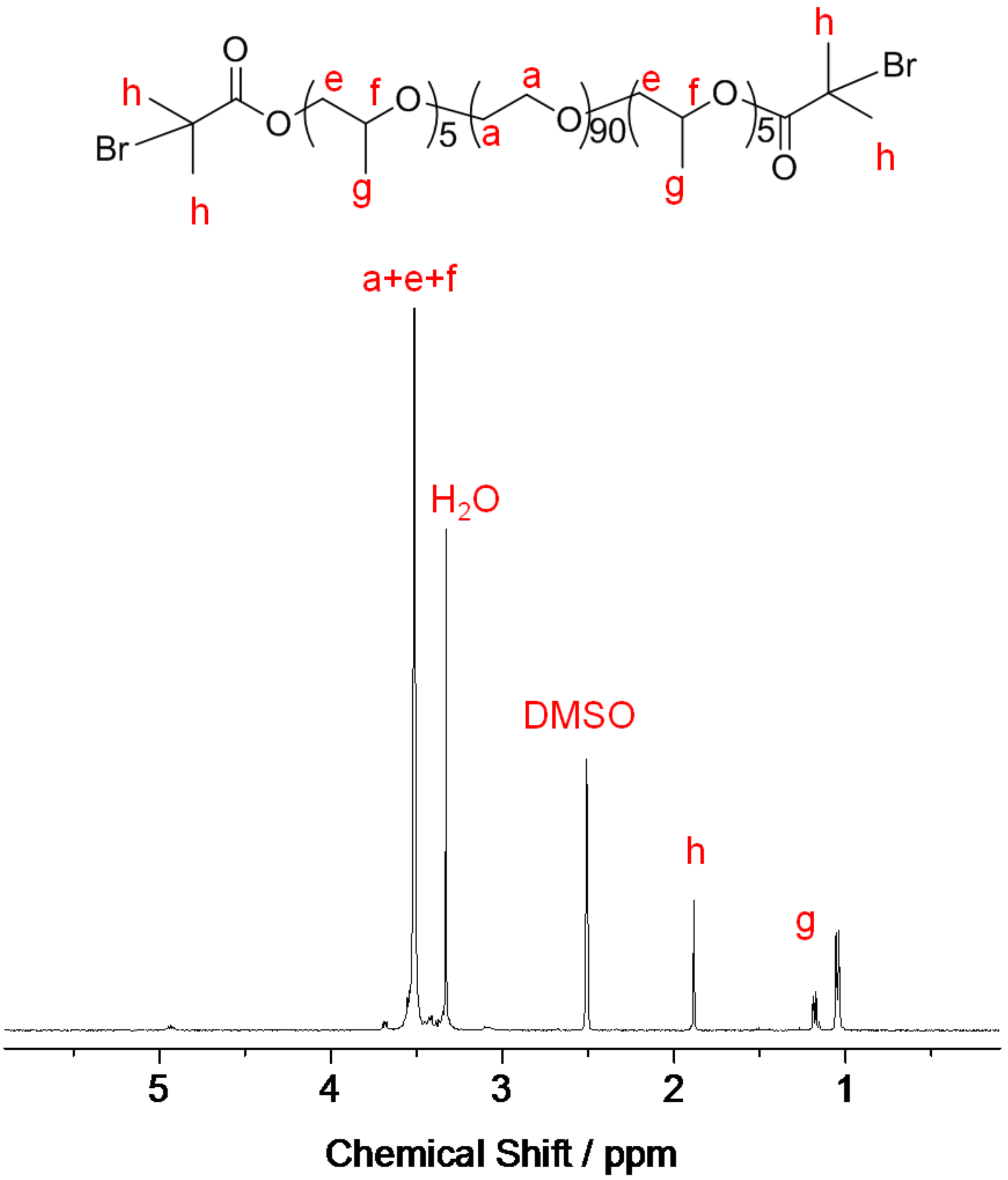

Figure S 3: ^{1}H NMR spectrum of BrPEPBr.
